# Methotrexate‑associated B‑cell lymphoproliferative disease that exhibits hematuria due to urinary bladder lesions: A case report

**DOI:** 10.3892/mi.2024.168

**Published:** 2024-06-12

**Authors:** Masahiro Manabe, Yuki Nagano, Takahiro Okuno, Takeshi Inoue, Ki-Ryang Koh

**Affiliations:** 1 Department of Hematology, Osaka General Hospital of West Japan Railway Company, Osaka 545-0053, Japan; 2 Department of Urology, Minamiosaka Hospital, Osaka 559-0012, Japan; 3 Department of Pathology, Minamiosaka Hospital, Osaka 559-0012, Japan; 4 Department of Pathology, Osaka City General Hospital, Osaka 534-0021, Japan

**Keywords:** B-cell lymphoproliferative disease, urinary bladder, methotrexate, hematuria, cystoscopy

## Abstract

Methotrexate (MTX)-related lymphoproliferative disease (LPD) is one of the most prominent late complications associated with MTX treatment. Although MTX-related LPD exhibits a relatively high incidence of extranodal disease, the incidence of disease in a urinary bladder is very low. The present study reports the case of a patient with MTX-related LPD involving a urinary bladder mass. A 75-year-old female patient, who had been receiving MTX for ~15 years, was referred to the hospital due to fever and hematuria. A computed tomography scan revealed the thickening of the urinary bladder wall, hydronephrosis and lymph node swelling. The histopathological findings of the urinary bladder mass resulted in a diagnosis of MTX-related LPD. Although MTX withdrawal did not have any effect, the subsequent chemotherapy resulted in complete remission. Although MTX-related LPD in the bladder is rare, it is pertinent to consider MTX-related LPD when hematuria is observed during MTX therapy.

## Introduction

Methotrexate (MTX) is currently widely used as a first-line treatment for rheumatoid arthritis (1). When malignant lymphoma develops during MTX treatment, the lymph node lesions often shrink when MTX is discontinued, and this condition is termed MTX-related lymphoproliferative disease (LPD) (2). Although MTX-related LPD is frequently associated with extranodal lesions, bladder lesions are rare in both lymphoma and MTX-related LPD (3,4). The present study reports the case of a patient with polymorphic B-cell LPD (B-LPD), who was human herpesvirus (HHV)8^-^ and Epstein-Barr virus (EBV)^-^, and iatrogenic (MTX), in which urinary bladder lesions developed.

## Case report

**Figure 1:**
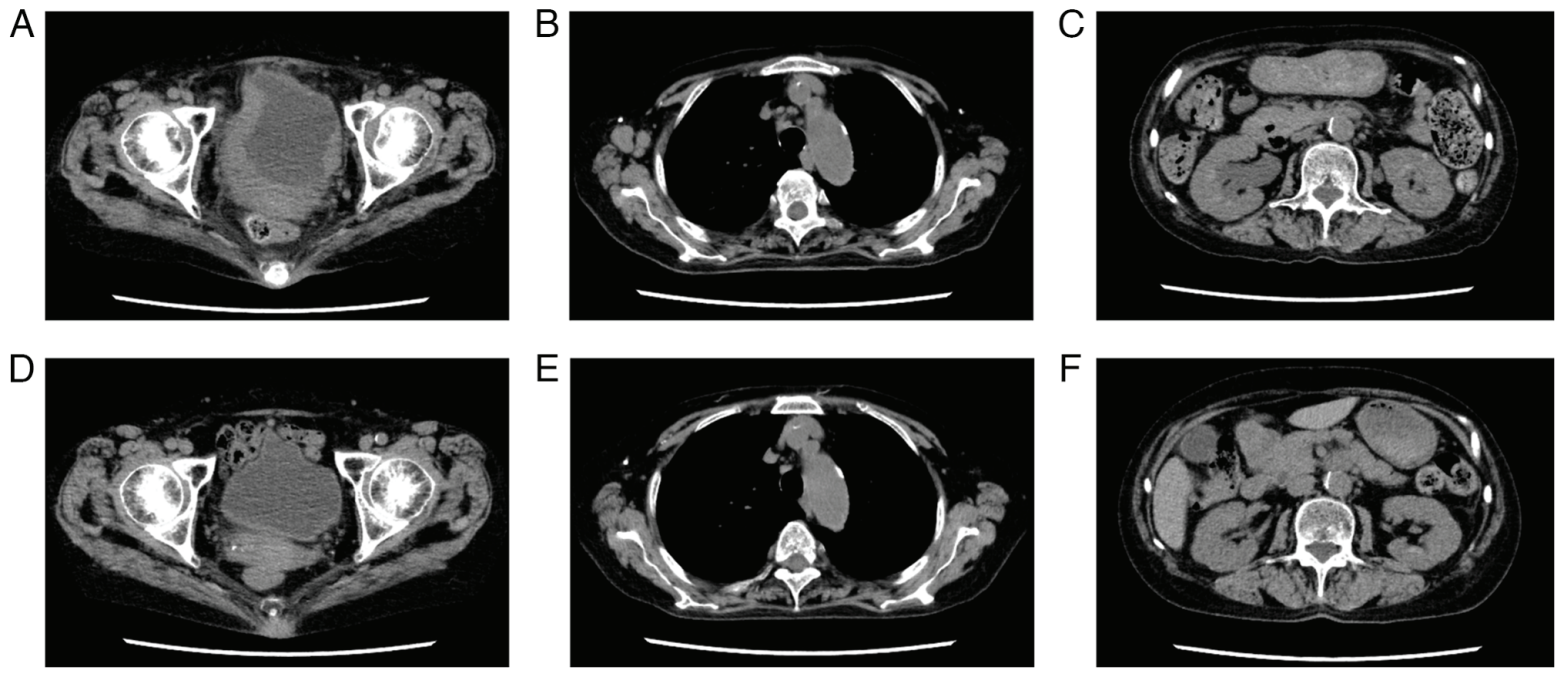
Computed tomography images demonstrating (A) irregular thickening of the urinary bladder wall, (B) swelling of the axillary and mediastinal lymph nodes, and (C) hydronephrosis. Following six cycles of chemotherapy, (D and E) the bladder wall thickening and lymph node swelling had disappeared, and (F) the hydronephrosis had improved.

**Figure 2:**
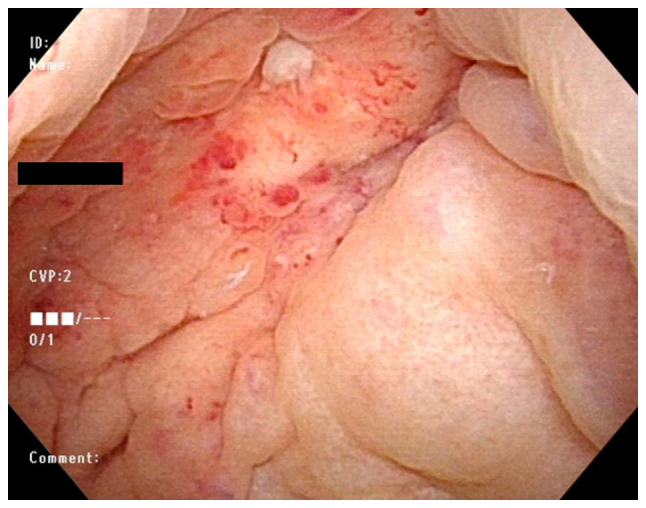
A cystoscopic image of the urinary bladder mass with a clot on the posterior wall.

**Figure 3:**
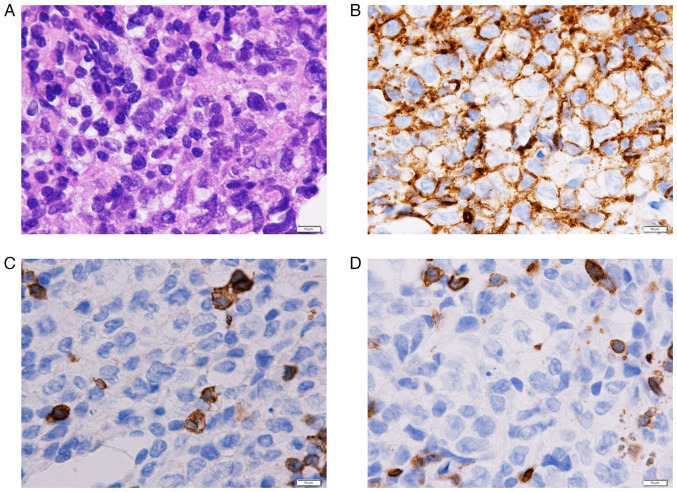
(A) Pathological images of the bladder specimen illustrating the diffuse proliferation of medium-to-large polymorphous lymphocytes with mild atypia (hematoxylin and eosin staining; magnification, x1,000). (B-D) Immunohistochemical staining demonstrated positivity for (B) CD20 (magnification, x1,000), and negativity for (C) CD3 (magnification, x1,000), and (D) CD5 (magnification, x1,000).

A 75-year-old female patient was referred to Minamiosaka Hospital (Osaka, Japan) due to fever and hematuria. She had a history of rheumatoid arthritis, which had been treated with 6 mg/week of MTX and 200 mg/day of bucillamine for ~15 years. She had no history of chronic cystitis. A physical examination revealed axillary lymph node swelling. Peripheral blood analysis revealed a white blood cell count of 14,300/µl, a hemoglobin concentration of 13.7 g/dl and a platelet count of 176,000/µl. Her serological levels were as follows: Lactate dehydrogenase, 260 U/l; C-reactive protein, 11.74 mg/dl; blood urea nitrogen, 18.3 mg/dl; creatinine, 0.70 mg/d; and soluble interleukin-2 receptor, 2,460 U/ml. Although urinalysis revealed macrohematuria (50-99 red blood cells per high power field) and *Escherichia coli* grew from her urine culture, a cytological examination was negative. A computed tomography scan revealed axillary, mediastinal and pelvic lymph node swelling, abnormal masses in the urinary bladder wall and hydronephrosis (Fig. 1). She underwent rigid cystourethroscopy to investigate the etiology of the macrohematuria. A large mass was identified in the posterior bladder wall (Fig. 2). Partial transurethral resection of the bladder mass was performed. A biopsy of the mass demonstrated the massive proliferation of medium-to-large polymorphic lymphoid cells with prominent nucleoli (Fig. 3). The mass blocks were fixed in a solution containing 10% formaldehyde (Yuaikasei Co., Ltd.) in 0.01 M phosphate-buffered saline (Muto Pure Chemicals Co., Ltd.) for 2 h at room temperature. Following fixation, the tissue blocks were loaded into the Tissue-Tek^®^ TEC 6 (Sakura Finetek Japan Co., Ltd.) and subsequently embedded in paraffin. Formalin-fixed paraffin-embedded sections were cut (3-µm-thick) using a YAMATO REM-710 microtome (Yamato Kohki Industrial Co., Ltd.). The subsequent dewaxing process included sequential treatments with xylene, anhydrous ethanol, a decreasing concentration gradient of ethanol, and water. Following this, the sections were immersed in hematoxylin staining solution (Muto Pure Chemicals Co., Ltd.) for 5 min at room temperature and then differentiated with 0.3% acid alcohol before being incubated with 0.6% ammonia for 1 min at room temperature. Eosin staining solution (Muto Pure Chemicals Co., Ltd.) was applied for 3 min, followed by dehydration with ethanol and xylene. Finally, the samples were mounted with neutral gum to prepare the slides. Immunohistochemical staining revealed that the atypical lymphocytes were positive for CD20 (cat. no. 518-110086; Roche Diagnostics), BCL6 (cat. no. 418181; Nichirei Bioscience) and multiple myeloma oncogene 1 (MUM1) (cat. no. 418411; Nichirei Bioscience) and negative for CD3 (cat. no. 413591; Nichirei Bioscience), CD5 (cat. no. 413251; Nichirei Bioscience), EBV latent membrane protein 1 (LMP1; cat. no. 518102524; Roche Diagnostics) (tested at Osaka City General Hospital; data not shown), and HHV8 (cat. no. 40-0198-32; Roche Diagnostics) (tested at Osaka City General Hospital; data not shown). Images were captured using a light microscope (BX43, Olympus Corporation) equipped with a DP73 camera (Olympus Corporation). Although diffuse large B-cell lymphoma (DLBCL) was considered as a differential diagnosis as the massive proliferation of atypical lymphocytes was observed, the atypical lymphocytes, whose sizes varied, had altered the architecture of the lymph nodes, which did not fulfill any of the criteria for DLBCL. Hence, a pathological diagnosis of polymorphic B-LPD, HHV8^-^, EBV^-^, iatrogenic (MTX) was made, according to the fifth edition of the World Health Organization classification of hematolymphoid tumors (5).

Although antibiotic treatment [Ceftriaxone (TAIYO Pharma Co., Ltd.) 1 g twice a day for 2 weeks] for a urinary tract infection was commenced, and MTX was discontinued, the patient's fever did not improve. Furthermore, the anemia caused by the hematuria gradually became more severe, and the hemoglobin concentration of the patient decreased to 8.6 g/d at 1 month following the initial diagnosis. In addition, a computed tomography scan revealed that the urinary bladder wall thickening had not improved. As regards the therapeutic approach, limited transurethral resection of the bladder tumor was considered to be difficult as wall thickening was widespread throughout the bladder. As MTX withdrawal alone was not effective, it was deemed necessary for chemotherapy to be commenced. Owing to the poor performance status of the patient (grade 3 according to the Eastern Cooperative Oncology Group performance status scale) the patient received reduced-intensity chemotherapy at Osaka General Hospital of West Japan Railway Company (Osaka, Japan), involving 375 mg/m^2^ rituximab (Kyowa Hakko Kirin Co., Ltd.) on day 1, 400 mg/m^2^ cyclophosphamide (Shionogi & Co., Ltd.) on day 2, 1 mg/body vincristine (Nippon Kayaku Co., Ltd.) on day 2, 25 mg/m^2^ doxorubicin (Sandoz K.K.) on day 2, and 40 mg/m^2^ prednisolone (Shionogi & Co., Ltd.) for 5 days (6). Following six cycles of the chemotherapy, a computed tomography scan demonstrated that the mass had disappeared (Fig. 1D-F). There has been no evidence of recurrence for >1 year since the initial diagnosis; the patient has remained free of any symptoms, including hematuria and fever; and her performance status has recovered to grade 1.

## Discussion

MTX has immunosuppressive effects when administered at low doses and is used in the treatment of autoimmune diseases. It is also used as an anchor drug for rheumatoid arthritis because it suppresses joint destruction (1). MTX-related LPD is one of the most prominent late complications of MTX treatment and is classified under the ‘other iatrogenic immunodeficiency-associated LPD (OII-LPD); category in the latest World Health Organization classification (5). As regards its characteristics, MTX-related LPD occurs in patients receiving MTX, and it is frequently complicated by extranodal lesions. In some cases, LPD lesions regress and remission is achieved when MTX is discontinued. Epidemiologically, rheumatoid arthritis is the most common underlying disease; female patients account for approximately two-thirds of cases (7-9). It has been reported that the median age at onset is 65 to 70 years, the median duration of the underlying disease is ≥10 years, and the median duration of MTX treatment is ≥5 years (7-9). In addition, a high EBV positivity rate has been reported, and it has been shown that 71.9% of cases of polymorphic LPD are positive for EBV (8). In the case in the present study, the disease was considered to be at an advanced stage, as the extranodal bladder lesions were causing macrohematuria, and chemotherapy was prioritized; hence, it was not possible to confirm that the lesions had been resolved by the discontinuation of MTX, as chemotherapy was initiated after only 1 month of MTX withdrawal. However, the background diseases, sex, age at onset and duration of MTX use were typical of MTX-associated LPD. Therefore, the patient in the present study was diagnosed with MTX-related LPD, even though the histopathological specimens of the patient demonstrated a negative EBV status.

As regards extranodal involvement, 40-50% of patients with MTX-related LPD have extranodal disease (2,3). A previous study examined extranodal disease among 89 patients with OII-LPD (3). The extranodal disease was located in the liver or spleen (10 patients each); the lungs or skin (7 patients each); the adrenal grand, pleura, or bone (3 patients each); the gingiva (2 patients); or the kidneys, small bowel, or breast (1 patient each) (3). In the present case, the presence of lesions in the urinary bladder at the initial diagnosis was consistent with MTX-related LPD. In both lymphoma and MTX-related LPD, invasion into the urinary bladder as an extranodal lesion is rare. Among primary lymphomas, invasion into the urinary bladder is only observed in ~0.2% of cases, and advanced or secondary bladder lymphoma accounts for 1.8% of secondary tumors of the bladder (4). This is mainly due to the lack of lymphoid tissue in the urinary bladder. Ohsawa *et al* (10) suggested that the mechanism by which lymphoma develops in the bladder, where there is no lymphoid tissue, may involve lymphocyte infiltration against a background of preexisting chronic inflammation. On the other hand, no evident history of cystitis was found in the case described herein. Similarly, cases without chronic inflammation have been previously reported (10-12), and therefore, uncertainty still exists regarding the role of chronic cystitis in the development of lymphoma in the bladder. Although bladder lesions developed as MTX-related LPD in the present case, only one case of OOI-LPD that caused lesions in the bladder has been reported to date, at least to the best of our knowledge (this involved a patient that had been treated with enzalutamide) (13), and there have not been any cases of MTX-related LPD involving urinary bladder disease reported in the English literature to date, at least to the best of our knowledge. As regards symptoms, lymphoid neoplasms of the urinary bladder present with hematuria, dysuria, urinary retention and urinary tract infections, regardless of the pathological subtype. Among these, hematuria is the most frequently observed symptom (14). Therefore, in the case that hematuria is observed in a patient treated with MTX, OOI-LPD of the bladder should be considered as a differential diagnosis.

As regards treatment, the main distinguishing feature of OII-LPD is spontaneous regression following the discontinuation of the causative immunosuppressant. A recent study reported that regression following MTX withdrawal was observed in 70% of patients with MTX-LPD (15). On the other hand, the remaining 30% of patients did not exhibit regression, even after MTX withdrawal. In addition, although the rapid regression of LPD following the discontinuation of MTX strongly suggests that there is a causative association between the drug and LPD, the median time to maximal efficacy following the cessation of MTX in cases in which chemotherapy was not administered was 12 weeks (range, 2-76) (16). Hence, it was proposed that chemotherapy should be performed without delay when LPD does not regress, or relapses or recurs, following MTX withdrawal (17). In another study, spontaneous regression following the discontinuation of MTX was observed in 23 (53.5%) of 43 patients with MTX-LPD who were followed-up, and EBV positivity was significantly higher in the spontaneous regression group (85.2%) than in the no regression group (50%) (3). In the case present herein, MTX discontinuation was not effective; this may have been due to the fact the disease was EBV-negative. Therefore, when selecting the optimal treatment strategy, it is necessary to consider the EBV status and progress of the disease to determine whether the discontinuation of MTX alone is acceptable or whether chemotherapy is necessary. As for chemotherapy, although the case in the present study was pathologically diagnosed with polymorphic LPD, its clinical course was relatively rapid; hence, a type of chemotherapy that is commonly uses to treat DLBCL was administered. As aforementioned, a reduced-intensity chemotherapeutic regimen designed for elderly patients was selected due to the poor performance status of the patient. However, this reduced-intensity regimen has recently been shown to be less effective than standard-dose chemotherapy (18). In addition, the survival of patients with urinary tract lymphoid neoplasms has not improved significantly over the past two decades despite the introduction of modern therapies, such as rituximab (19). Recently, chemotherapeutic regimens, including polatuzumab have been reported to exhibit superior efficacy to the R-CHOP (rituximab plus cyclophosphamide, doxorubicin, vincristine and prednisone) regimen in patients with treatment-naïve DLBCL aged 18-80 years (20). Furthermore, a phase III clinical study comparing a reduced-intensity R-CHOP regimen with reduced-intensity polatuzumab-containing chemotherapy for elderly patients is currently in progress (NCT04332822, https://clin.larvol.com/abstract-detail/EHA%202023/65463867). Hence, it is suggested that a therapeutic strategy, including polatuzumab should be considered for the treatment of patients with OOI-LPD, particulalry in elderly patients and/or patients with poor performance statuses for whom MTX withdrawal is not effective.

In conclusion, the presented study describes the case of a patient with MTX-related LPD accompanied by extranodal bladder lesions. Although lymphoid invasion into the urinary bladder as an extranodal lesion is rare, it is critical for clinicians to be aware that MTX-related LPD in the bladder can occur as a late complication of MTX treatment. As regards therapeutic interventions, MTX discontinuation may not be effective, depending on patient factors, such as EBV negativity. Hence, when MTX withdrawal fails, it is necessary to make an appropriate decision regarding whether it is optimal to treat the patient by persisting with MTX discontinuation alone or plan for chemotherapy.

## Data Availability

The datasets used and/or analyzed during the present study are available from the corresponding author upon reasonable request.
